# Unfolded protein response during the progression of colorectal carcinogenesis

**DOI:** 10.1590/acb400725

**Published:** 2025-01-13

**Authors:** Cesar Augusto, Alexandre Moreira de Almeida, Maytana Roberta Taschin Grigolo, Michele Selzler, Antônio Carlos de Abreu, Letícia Silva Fagundes, Almir Souza Martins, Durval Batista Palhares, Rondon Tosta Ramalho

**Affiliations:** 1 Universidade Federal de Mato Grosso do Sul – Postgraduate Program in Health and Development in the Midwest Region – Campo Grande (MS) – Brazil.; 2 Instituto de Assistência à Pesquisa em Educação e Saúde – Campo Grande (MS) – Brazil.

**Keywords:** Colorectal Neoplasms, Endoplasmic Reticulum Stress, Unfolded Protein Response

## Abstract

**Purpose:**

To evaluate the molecular evolution of endoplasmic reticulum (ER) stress during colorectal cancer carcinogenesis.

**Methods:**

Fifty-six hairless mice were divided into two groups: control (no intervention); and carcinogenesis (treated with two doses of azoxymethane at 10 mg/kg during the third and the fourth week and dextran sodium sulfate at 2.5% for seven days in the second, fifth, and eighth week). Euthanasia occurred at the fifth, 10th, 15th, and 20th week. Colons were collected, and gene expression of ER stress markers (IRE1-α, PERK, ATF6, and CHOP) was assessed via real-time quantitative polymerase chain reaction.

**Results:**

ERN1 expression was significantly higher than the control at the 10th week (1.39 ± 0.16, *p* 0.05) and the 20th week (1.15 ± 0.04, *p* 0.01). ATF6 also showed elevated expression, significantly different at the 10th week (1.71 ± 0.29, *p* 0.05) and the 20th week (1.14 ± 0.06, *p* 0.05). PERK and CHOP expressions were significantly higher than the control in the 15th (PERK = 1.30 ± 0.12, CHOP = 1.48 ± 0.23) and 20th weeks (PERK = 1.63 ± 0.20, CHOP = 1.67 ± 0.22, *p* 0.05).

**Conclusion:**

Upregulation of IRN1, PERK, ATF6, and CHOP demonstrates a strong ER stress response during colorectal cancer development.

## Introduction

Colorectal cancer (CRC) is a common and lethal disease. It is the third most prevalent malignant tumor of the gastrointestinal tract, but it holds the second position in fatality rates. It is the third most prevalent cancer in men, following lung and prostate cancer, and the second most prevalent cancer in women, after breast cancer. Projections indicate that the worldwide incidence of CRC would approach 2.2 million new cases annually by 2030, reflecting an additional 20% rise^1^.

Most CRCs originate from polyps, which are benign, flat, or elevated lesions that serve as precursors to CRC and arise in the mucosa of the large intestine’s inner wall^2^. The primary risk factors for CRC include a familial history of bowel cancer, a personal history of bowel cancer, age 50 or older, obesity, a diet high in fats, sugars, and processed meats while low in fiber, physical inactivity, diabetes mellitus, tobacco use, and alcohol consumption, among others^3^.

In eukaryotic species, the endoplasmic reticulum (ER) is a crucial organelle that plays a variety of vital roles, including protein synthesis, folding, and modification. It also serves as a calcium reservoir and the primary compartment for lipid production^4^.

The unfolded protein response (UPR) is a crucial biological process for preserving protein folding equilibrium in the ER^5^. When misfolded proteins accumulate in the ER, the UPR is activated to enlist chaperone proteins that aid in rectifying the aberrant proteins. This mechanism alleviates stress on the ER by diminishing the quantity of misfolded proteins, enhancing the ER’s folding capacity, and destroying proteins that cannot be promptly refolded. Upon the alleviation of protein stress by the UPR, it is deactivated, restoring cellular equilibrium to its usual state. Should the UPR prove ineffective in alleviating the stress, the cell may be compelled to undergo apoptosis^6^^,^ 7.

The UPR comprises three principal transmembrane proteins: inositol-requiring enzyme 1-α (IRE1α), PERK-like ER kinase (PERK), and activator of transcription factor 6 (ATF6), which orchestrate signaling cascades to restore ER homeostasis and save the cell, or to initiate death if the stress is deemed irreversible. These proteins operate interdependently, activating and inhibiting molecules in the endoplasmic reticulum, cytosol, and nucleus^7^. The ER equilibrium is crucial for the proper functioning of cellular activities. ER stress (ERS) can be triggered by conditions such as increased demand for protein secretion or synthesis, and it is implicated in the development of various pathologies, including cancer, in which the tumor microenvironment (TME) necessitates elevated levels of oncoproteins that constitute the architecture of neoplastic tissue^8^. The UPR, upon activation, initiates cytoplasmic and nuclear signaling that promotes the refolding of malfunctioning proteins, their degradation, a decrease in protein synthesis, or, in severe cases, cellular apoptosis^9^.

Tumor formation is influenced by UPR signaling, and it is widely acknowledged that ERS and UPR activation plays a role in the initiation and spread of numerous malignancies, including CRC^10^. The CRC is recognized for its multifactorial pathophysiology and is intricately linked to ERS associated with the UPR, which encompasses the primary cytomolecular pathways implicated in the development, progression, and invasion of colon tumors^1^.

The C/EBP homologous protein (CHOP) is a crucial modulator of ERS-induced apoptosis in the PERK signaling cascade. A key route that often leads to ERS apoptosis is the signaling sequence PERK - IRE1α - ATF6 - CHOP. Furthermore, early in the ERS process, ATF6 plays a significant role in the synthesis of CHOP^11^.

In the TME, ERS influences the development of tumors and the activity of immune cells. Uncertainty persists regarding the fundamental function of ERS-related genetic patterns in the development of CRC^12^. Based on this, the study aimed to evaluate the molecular evolution of ERS during CRC carcinogenesis.

## Methods

All procedures were approved under no. 1,204/2022 on February 10, 2022, and were registered with the Animal Use Ethics Committee of the Universidade Federal de Mato Grosso do Sul (UFMS), following the ethical guidelines set forth by the National Council for the Control of Animal Experimentation.

### Research location

Planning and development occurred at the Experimental Carcinogenesis Laboratory; the implementation of the animal model took place at the Laboratory of Studies in Stem Cells, Cell Therapy, and Toxicological Genetics (CeTroGen); and the processing and analysis of samples were conducted at the Molecular Pathology Laboratory, all affiliated with the School of Medicine of UFMS.

### Animals

Fifty-six male inbred HRS/J mice, designated as hairless, aged 4 weeks old and weighing approximately 25 g, were obtained from the Central Bioterium of the Institute of Biosciences at the UFMS Foundation. Prior to the commencement of the experiment, the animals were acclimatized in the laboratory, provided with water and regular chow *ad libitum* for 14 days. During the experiment, the animals were housed on two ventilated shelves, maintained at a controlled temperature of 23°C, with artificial white lighting on a 12-hour light-dark cycle.

### Experimental design

The animals were divided into two groups, comprising 28 animals total, with four boxes per group containing seven animals each:

- G1 (control): they were provided with only water and standard food *ad libitum*;
- G2 (carcinogenesis): administered two doses of azoxymethane at 10 mg/kg during the third and fourth week, and dextran sodium sulfate at 2.5% for seven days in the second, fifth, and eighth week.

**Figure 1:**
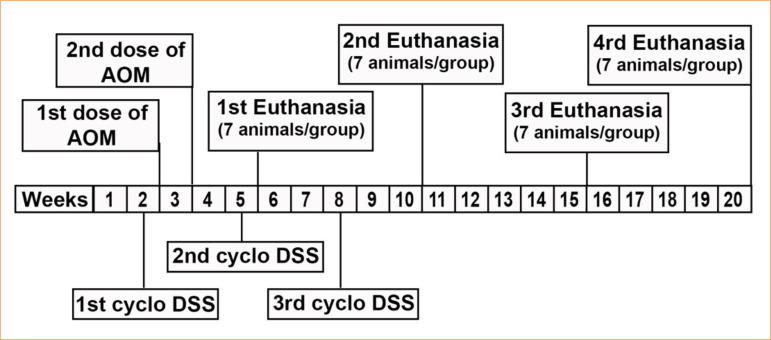
Experimental design: timeline.

Source: Elaborated by the authors.

The animals were assessed daily for indications of illness or distress and were weighed prior to the commencement of the experiment and weekly during the study, with the objective of identifying and tracking changes that could affect the weight loss or growth of each animal. Euthanasia was conducted during the fifth, 10th, 15th, and 20th weeks. Figure 1 illustrates the structure of the experimental investigation and its distinct phases over time.

### Confirmation of carcinogenesis

The progression of colonic carcinogenesis was assessed through the identification of polyps and histological changes in the distal colon of the animals with hematoxylin and eosin staining.

### Total RNA extraction

The colon segments from each animal were maintained at -80ºC in separate cryovials, and the genomic quantification of mRNA for the proteins IRE1-α, PERK, ATF6, and CHOP was conducted at four intervals (the end of the fifth, 10th, 15th, and 20th week of the experiment).

Fragments of tissue samples from the distal colon segment, weighing approximately 200 mg, were subdivided into 100-mg portions and immersed in RNAlater™ stabilization solution. These were stored at -20ºC for a brief duration before undergoing total RNA extraction via the TRIZOL method, in accordance with the manufacturer’s protocol.

### Primers

Primers for reverse transcription (RT) and real-time quantitative polymerase chain reaction (qPCR) were developed with public mRNA sequences from GenBank, employing BLASTn software (www.ncbi.nlm.nih.gov/blast/blast.cgi), and the qPCR Assay Design-PrimerQuestTM tool from Integrated DNA Technologies (IDT; http://www.idtdna.com). The S26 mRNA target served as the endogenous normalizer (Table 1).

**Table 1:** Primers for IRE1-α, PERK, ATF6, CHOP and S26.

Primers	Nucleotide sequence	Size	Amplified fragment
MMIRE1-αFOR MMIRE1-αREV	5´ – CCA GCA CAG ACC TCA AGT TT – 3´ 5´ – GAG GAG TTT CAT GGT GTC CTA TG – 3´	20 nt 23 nt	99 pb
MMPERKFOR MPERKREV	5’ – ACC GGG TGG AAA CAA AGA A – 3’ 5’ – CTT CCA ATC AGC AAC GGA AAC – 3’	19 nt 21 nt	103 pb
MMATF6FOR MATF6REV	5´ – CAG AGA ACC AGA GGC TCA AA – 3´ 5´ – GGG CCC ATA GTT CAG CAT TA – 3´	20 nt 20 nt	92 pb
MMCHOPFOR MMCHOPREV	5´ – TCA CAC GCA CAT CCC AAA – 3´ 5´ – CCT AGT TCT TCC TTG CTC TTC C – 3´	18 nt 22 nt	93 pb
MMS26FOR MMS26REV	5' – CGT GCT TCC CAA GCT CTA TGT – 3' 5' – CGT GCT TCC CAA GCT CTA TGT – 3'	21 nt 21 nt	75 pb

nt: nucleotode; pb: base pairs. Source: Elaborated by the authors.

### Reverse transcription and real-time polymerase chain reaction

Synthesis of single-stranded complementary DNA (sscDNA) was conducted via RT, utilizing 700 ng of DNase I-treated RNA (following the TURBO DNA-free kit protocol, Ambion Inc., Foster, California, United States of America). The RNA was preincubated at 70°C for 10 minutes with 10 pmol of each reverse primer and 10 pmol of oligo dT20 primer (Invitrogen), subsequently stored on ice at room temperature. Subsequently, 40 U (11 µL) of reverse transcriptase enzyme mix in RT buffer (50 mM KCl, 20 mM Tris-HCl, pH 8.4), supplemented with 2 µL of dNTP mix (10 mM each), was incubated at 45°C for 1 hour alongside the RNA and primer solution. RT was halted at 4°C and promptly utilized in qPCR.

sscDNA samples were utilized in qPCR conducted on the QuantStudio 6 Flex Real-Time System (ThermoFisher Scientific), following the reaction methodology outlined by the manufacturer (Invitrogen Life Technologies, Carlsbad, CA, United States of America; SYBR Green PCR Master Mix Kit). Duplicate samples were dispensed into 384-well plates (ABI PRISM 384-Well Optical Reaction Plate with Barcode, Invitrogen Life Technologies, Carlsbad, CA, United States of America), with a final reaction volume of 10 µL per sample. Two µL of sscDNA from the samples were dispensed into each microwell plate, accompanied by 8 µL of SYBR Mix, which comprised 5 µL of SYBR Green PCR Master Mix Kit, 0.6 µL of each primer (sense and antisense; 10 pmol/µl), and 1.8 µL of sterile filtered water. The plate was sealed using optical adhesive (ABI PRISM Optical Adhesive Covers, Invitrogen Life Technologies, Carlsbad, CA, United States of America).

Relative quantification involved comparing the expression of target gene transcripts (IRE1-α, PERK, ATF6, and CHOP) with the endogenous control (S26) using the comparative CT method. The endogenous control normalized the expression of the target genes by calculating the ΔCT (mean CT of the target gene minus mean CT of the endogenous control). The ΔΔCT was computed using the ΔCT, defined as [ΔCT of the sample - ΔCT of the calibrator (reference sample)]. The formula 2^–ΔΔCT^ was utilized to determine the relative expression levels (fold change in gene expression) of each target gene^13^.

### Statistic analysis

Statistical analyses were conducted utilizing GraphPad software version 5.00 (GraphPad Software Inc., San Diego, CA, United States of America), with a significance threshold established at *p* < 0.05 for all evaluations. The data underwent the Shapiro-Wilk’s normality test. Comparisons between two independent samples were conducted using the unpaired t-test or the Mann-Whitney’s test.

## Results

### Confirmation of carcinogenesis

**Figure 2:**
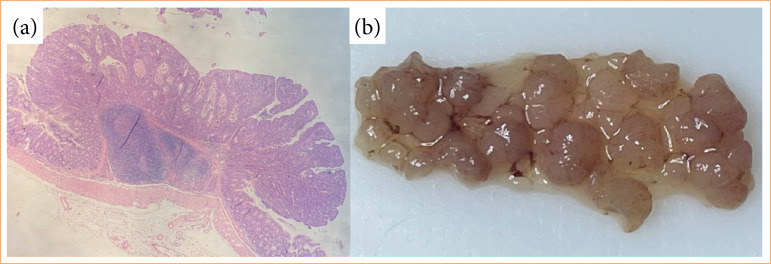
Presence of polyps and histological changes. **(a)** Histological changes on distal colon; **(b)** polyps at the distal region of the colons.

Source: Elaborated by the authors.

Carcinogenic progression was validated post-euthanasia through the presence of polyps and histological changes (including aberrant crypts, adenomas, and low- and high-grade dysplasia) identified with hematoxylin and eosin staining in the distal colon region (Fig. 2).

Expression of mRNA of IRE1-*α*, PERK, ATF6, and CHOP proteins

Table 2 displays the comparative expression levels of ERN1, PERK, ATF6, and CHOP mRNA in the carcinogenesis group versus the control group at the fifth, 10th, 15th, and 20th weeks.

**Table 2:** Relative expression of ERN1, PEK, ATF6 and CHOP mRNA in the fifth, tenth, 15th, and 20th week of the experiment in the carcinogenesis group in relation to the control group.

	ERN1	ATF6	PERK	CHOP
5th week	0.94 ± 0.05	0.99 ± 0.08	0.96 ± 0.07	0.74 ± 0.17
10th week	1.39 ± 0.16*	1.71 ± 0.29*	2.45 ± 0.53	1.56 ± 0.39
15th week	1.09 ± 0.03	1.11 ± 0.06	1.30 ± 0.12*	1.48 ± 0.23*
20th week	1.15 ± 0.04**	1.14 ± 0.06*	1.63 ± 0.20*	1.67 ± 0.22*

* *p* < 0.05 and

** *p* < 0.01 compared to control.

Data presented as mean ± standard error of the mean.

**Figure 3:**
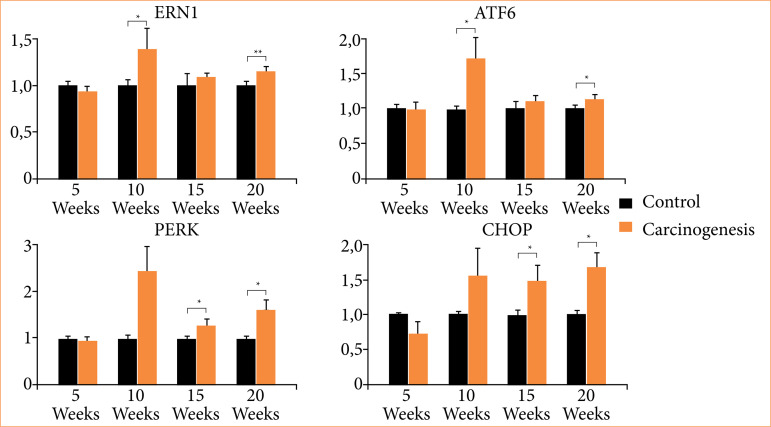
Relative expression of ERN1, PEK, ATF6 and CHOP. Relative expression of ERN1, PEK, ATF6 and CHOP mRNA in the 5th, 10th, 15th and 20th week of the experiment in the carcinogenesis group in relation to the control group.

ERN1 exhibited elevated expression compared to the control, with a significant difference at the tenth week (*p* < 0.05) and a highly significant difference at the 20th week (*p* < 0.01). ATF6 exhibited elevated expression compared to the control, with a statistically significant difference (*p* < 0.05) observed in the tenth and 20th weeks. Similarly, PERK and CHOP demonstrated increased expression relative to the control, with significant differences (*p* < 0.05) noted in the 15th and 20th weeks (Fig. 3).

*p < 0.05; **p < 0.01. Source: Elaborated by the authors.

## Discussion

Normal cells maintain the UPR in an inactive state under basal conditions, activating it solely in response to physiological stressors that impact the ER. This activation can facilitate both adaptation and survival, as well as apoptosis in tumor cells, while also affecting metastasis. UPR activation is essential for adaptation to hypoxia, nutrient scarcity, the generation of reactive oxygen species (ROS), and the functioning of the immune system^7^. On the other hand, cancer cells frequently activate the UPR to adapt and modulate anti-apoptotic signals, gaining the ability for self-selection and survival in unfavorable environments; the activation of PERK via ARE, Keap1, and ATF4 promotes survival in conditions of nutrient deficiency, ATP shortage, ROS production, and hypoxia, while the modulation of IRE1α induces the expression of XBP1, XBP1, and ERAD, aiding in the survival of malignant cells^7^.

Colorectal carcinogenesis is a multifaceted process that entails the interplay of various molecular pathways, including the ERS response. The expression of genes including ERN1 (IRE1), PERK, ATF6, and CHOP is essential in this process^10^. The synchronized activation of ERN1, PERK, ATF6, and CHOP during the weeks of carcinogenesis indicates a cohesive process in which the early ERS response is primarily characterized by adaptation and restoration of homeostasis, then transitioning to a phase of apoptosis induction when stress becomes chronic^14^. The notable elevations in PERK and CHOP expression at the 15th and the 20th week suggest an essential shift from a protective mechanism to an apoptotic response, crucial for averting the unchecked proliferation of injured cells. The findings align with the current literature, indicating that sustained activation of the ERS response is frequently linked to tumor development and resistance to treatment. Therapeutic approaches that adjust the response to ERS may enhance treatment results for CRC, particularly in later stages^15^^–^ 17.

Inositol-requiring enzyme 1-alpha (IRE1-α) is an ERS sensor that activates UPR signaling via the splicing of XBP1 mRNA, which encodes a transcription factor that facilitates the expression of UPR target genes. It is upregulated in CRC, aiding tumor cell survival and proliferation by augmenting adaptive responses to ERS^18^. Activation of IRE1-α has been linked to enhanced cell viability and apoptosis resistance in CRC cells^19^. ERN1 expression exhibited a notable increase at week 10 and reached a highly significant level at week 20 in comparison to the control group. This increase indicates an initial activation of the IRE1 pathway, a principal sensor of the ERS response. Activation of IRE1 facilitates the processing of XBP1 mRNA, culminating in the synthesis of a protein that contributes to the reestablishment of ER homeostasis^20^. Research suggests that sustained activation of IRE1 may enhance cell survival during the initial phases of carcinogenesis; nevertheless, its extended activation may trigger apoptosis^21^.

PERK functions as a critical ERS sensor that phosphorylates eIF2α, resulting in a transient decrease in protein synthesis and the activation of ATF4. Research demonstrates that PERK signaling is essential for the adaptability of CRC cells to the challenging tumor microenvironment. PERK activation is linked to enhanced cell survival, proliferation, and treatment resistance in CRC^1^. Numerous studies have documented the upregulation of PERK signaling in CRC, underscoring its contribution to tumor progression and therapeutic resistance^20^. In this study, PERK expression exhibited a substantial rise at the 15th and the 20th week. PERK is activated in response to the accumulation of misfolded proteins in the ER and influences eIF2α phosphorylation, resulting in a reduction of global protein translation and alleviating ER stress; however, sustained activation of PERK can induce CHOP, a pro-apoptotic protein, indicating that the PERK pathway may initially safeguard cells from stress, but, over time, facilitate apoptosis^21^^,^ 22.

In reaction to ERS, ATF6 undergoes cleavage, releasing a cytosolic component that functions as a transcription factor to activate UPR target genes. Researchers have demonstrated that activating ATF6 in CRC enhances cell survival by upregulating genes associated with protein folding and degradation. CRC tissues exhibit elevated ATF6 expression, suggesting its involvement in tumor growth and ER adaptability^23^. According to previous research, ATF6 moves to the nucleus when it is activated and acts as a transcription factor for genes that control protein folding and degradation in the ER. The initial response to ERS correlates with its activation, enhancing cellular adaptive capacity. In CRC, it is associated with heightened proliferation and diminished apoptosis, underscoring its significance in cancer progression^24^. In persistent stress situations, ATF6 signaling may be inadequate for restoring homeostasis, leading to cellular malfunction and death^25^^,^ 26. In our study, ATF6 levels rose significantly at the 10th and the 20th week, indicating that the cell’s adaptive capacity during the evolution of CRC led to increased proliferation.

CHOP’s role in promoting apoptosis under unresolved ERS is well documented. The significant increase in CHOP expression at later stages of carcinogenesis indicates the transition from adaptive to apoptotic responses in CRC cells, resulting cell apoptosis. Research indicates that CHOP serves a dual function in CRC: it can induce apoptosis in response to significant ERS, while its temporary activation may facilitate the adaptability and survival of tumor cells under moderate stress circumstances^23^. CHOP exhibited a notable rise at the 15th and the 20th week, signifying the progression from adaptive to apoptotic responses in CRC cells during the advanced phases of carcinogenesis. CHOP is a crucial modulator of ERS-induced apoptosis. The expression is triggered by the activation of PERK and ATF6 and is linked to the facilitation of apoptosis due to prolonged and unresolved ERS. The equilibrium between CHOP-induced apoptosis and adaptive UPR signaling is essential in influencing the destiny of CRC cells during ERS^1^^,^ 22.

The interaction among these UPR components and their collective impact on CRC development underscores the intricacy of ERS signaling in cancer. For instance, interaction among the IRE1-α, PERK, and ATF6 pathways can either collaborate to promote cell survival or antagonize one another to trigger apoptosis, contingent upon the stress setting^24^. Recent investigations indicate that IRE1-α and PERK inhibitors can diminish CRC cell viability and enhance the effectiveness of standard therapies^20^. This complex equilibrium highlights the potential of targeting UPR components for therapeutic intervention in CRC through techniques designed to modulate UPR signaling, which are being investigated to enhance CRC therapy outcomes. Targeting certain UPR components, including IRE1-α, PERK, and ATF6, can alter the adaptive responses of CRC cells to ERS, hence enhancing their sensitivity to chemotherapy and facilitating death^1^.

This study offers several considerations that may be further developed in future research. While the experimental paradigm utilizing hairless mice is broadly recognized in carcinogenesis research and has produced significant findings, it may not entirely replicate the intricacies of colorectal carcinogenesis in humans. Moreover, incorporating complementary proteomic analysis could enhance the comprehension of protein dynamics related to ERS during tumor progression. While the study concentrated on the gene expression of four pivotal proteins (IRE1, PERK, ATF6, and CHOP), investigating additional molecular pathways may yield a more holistic understanding of the interplay between stress responses and cancer growth. Subsequent research could further elucidate the translation of these findings into a clinical environment, with the objective of identifying possible therapeutic targets for CRC treatment.

## Conclusion

The coordinated upregulation of IRN1, PERK, ATF6, and CHOP shows that the ERS response is strong in the development of CRC. Initially, the UPR attempts to mitigate stress through adaptive mechanisms, as indicated by increased IRN1, PERK, and ATF6. However, persistent stress leads to the activation of apoptotic pathways, marked by elevated CHOP levels. This dynamic response reflects the dual role of the UPR in promoting cell survival and inducing apoptosis depending on the severity and duration of ERS.

## Data Availability

The data will be available upon request.
